# Supplementary material for: Early Trauma Leaves No Social Signature in Sanctuary-Housed Chimpanzees (Pan troglodytes)
Source: Animals (Basel). 2022 Dec 22;13(1):49. doi: 10.3390/ani13010049 (PMC9817851; doi:10.3390/ani13010049)
Supplement: Supplementary file 1 [file animals-13-00049-s001.zip › animals-1980759-supplementary.pdf]

Supplementary Materials for

Early Trauma Leaves No Social Signature in Sanctuary-Housed Chimpanzees (*Pan troglodytes*)

Table S1. Demographic details of the chimpanzees under study.

Population	Subject	Age*	Sex	Dam	Origin	Subspecies
1	Pal	32,0	male	unknown	wild	schweinfurthii
1	Booboo	31,0	male	unknown	wild	schweinfurthii
1	Girly	31,0	female	unknown	wild	schweinfurthii
1	Tobar	31,0	male	unknown	wild	verus
1	Rita	30,0	female	unknown	wild	schweinfurthii
1	Tara	30,0	male	unknown	wild	schweinfurthii
1	Ingrid	22,0	female	Liza	captive	schweinfurthii
1	Brenda	17,7	female	Bella	captive	schweinfurthii
1	Genny	16,0	female	Girly	captive	schweinfurthii
1	Renate	16,0	female	Rita	captive	schweinfurthii
1	Bob	11,9	male	Big Jane	captive	schweinfurthii
1	Gerard	10,9	male	Girly	captive	not determined
1	Ilse	10,8	female	Ingrid	captive	not determined
1	Regina	7,3	female	Renate	captive	not determined
1	Rusty	6,4	male	Rita	captive	not determined
1	Chrissie	6,3	female	Cleo	captive	not determined
1	Innocentia	6,2	female	Ingrid	captive	not determined
1	BJ	5,8	female	Big Jane	captive	not determined
1	Gonzaga	5,2	male	Genny	captive	not determined
1	Irene	1,1	female	Ingrid	captive	not determined
1	Rachel	0,7	female	Renate	captive	not determined
2	Noel	36,0	female	unknown	wild	schweinfurthii
2	Donna	29,0	female	unknown	wild	schweinfurthii
2	Coco	28,0	female	unknown	wild	schweinfurthii
2	Little Jane	28,0	female	unknown	wild	schweinfurthii
2	Maggie	27,0	female	unknown	wild	schweinfurthii
2	Misha	25,0	female	unknown	wild	schweinfurthii
2	Dora	24,0	female	unknown	wild	schweinfurthii
2	Pan	24,0	male	unknown	wild	schweinfurthii
2	Pippa	24,0	female	unknown	wild	schweinfurthii
2	Trixie	23,0	female	unknown	wild	schweinfurthii

2	Zsabu	23,0	male	unknown	wild	schweinfurthii
2	Diana	22,0	female	unknown	wild	schweinfurthii
2	Masya	22,0	female	unknown	wild	schweinfurthii
2	Violet	22,0	female	unknown	wild	schweinfurthii
2	Little Judy	18,0	female	Little Jane	captive	not determined
2	Dolly	16,6	female	Dora	captive	schweinfurthii
2	Carol	16,4	female	Coco	captive	not determined
2	Nikkie	15,7	female	Noel	captive	schweinfurthii
2	Mikey	15,0	male	unknown	wild	schweinfurthii
2	Tess	15,0	female	Tina	captive	not determined
2	Tilly	12,2	female	Trixie	captive	not determined
2	Maxine	11,8	female	Misha	captive	not determined
2	David	11,5	male	Diana	captive	not determined
2	Debbie	11,3	female	Donna	captive	not determined
2	Claire	10,8	female	Coco	captive	not determined
2	Doug	10,2	male	Dora	captive	not determined
2	Nina	10,0	female	Noel	captive	not determined
2	Vis	8,9	male	Violet	captive	not determined
2	Daisey	8,4	female	Diana	captive	not determined
2	Mary	7,4	female	Masya	captive	not determined
2	Long John	7,3	male	Little Judy	captive	not determined
2	Max	6,6	male	Misha	captive	not determined
2	Little Jenkins	6,2	female	Little Jane	captive	not determined
2	Moyo	5,6	male	Maggie	captive	not determined
2	Dizzy	5,4	female	Diana	captive	not determined
2	Charity	5,2	female	Carol	captive	not determined
2	Little Jones	2,7	male	Little Jane	captive	not determined
2	Little Jacky	1,0	male	Little Judy	captive	not determined
2	Martin	0,9	male	Misha	captive	not determined
2	Danny	0,9	male	Dora	captive	not determined
2	May	0,4	female	Maggie	captive	not determined
3	Buffy	28	female	unknown	wild	schweinfurthii
3	Sampie	21	male	unknown	wild	schweinfurthii
3	Clement	20	male	unknown	wild	schweinfurthii
3	Brian	19	male	unknown	wild	schweinfurthii
3	Barbie	18	female	unknown	wild	schweinfurthii
3	E.T.	18	female	unknown	wild	schweinfurthii
3	Roxy	18	female	unknown	wild	schweinfurthii
3	Junior	18	male	unknown	wild	schweinfurthii
3	Lori	18	female	Liza	captive	not determined
3	Louise	16	female	unknown	wild	schweinfurthii[#]
3	Bussie	9	male	Barbie	captive	not determined
3	Erika	7	female	E.T.	captive	not determined

3	Bruce	3,3	male	Barbie	captive	not determined
3	Lods	2,8	female	Lori	captive	not determined
4	Nicky	22	male	unknown	wild	schweinfurthii
4	Bobby	20	male	unknown	wild	schweinfurthii
4	Sinkie	19	male	unknown	wild	schweinfurthii
4	Julie	19	female	unknown	wild	schweinfurthii
4	Kambo	17	female	unknown	wild	schweinfurthii
4	Kathy	14	female	unknown	wild	schweinfurthii
4	Val	13	male	unknown	wild	schweinfurthii
4	Berta	13	female	unknown	wild	schweinfurthii[#]
4	Miracle	12,7	female	Maggie	captive	schweinfurthii
4	Commander	12	male	unknown	wild	schweinfurthii
4	Kit	8	male	Kambo	captive	not determined
4	Jack	4,9	male	Julie	captive	not determined
4	Ken	1,8	male	Kambo	captive	not determined

* At the end of the study (March 2013)
# Conflicting results, possibly "verus"
